# Mutational analysis of H5N1 hemagglutinins: identification of molecular determinants for efficient packaging into pseudotyped lentiviral particles

**Published:** 2011-01-10

**Authors:** DJ Tang, YM Lam, CH Lam, SL Chu, R Bruzzone, B Nal

**Affiliations:** 1 HKU-Pasteur Research Centre, Hong Kong, Hong Kong SAR

## 

Due to the high pathogenicity of H5N1 viruses, we have developed and characterized lentiviral particles pseudotyped with HA (H5pp) from a Cambodia H5N1 isolate, which can be used as a safe tool for high-throughput serological studies without the requirement of BSL-3 facilities. However, not all H5 HAs give rise to efficient production of H5pp. The main objective of this study is to understand the intrinsic properties of H5cam and H5anh (derived from A/Cambodia/2005/40808 and A/Anhui/2005/01 respectively) which exhibited a dramatic difference in their abilities to generate H5pp; and to identify molecular determinants that control the assembly and release of H5 pseudotyped lentiviral particles.

H5cam and H5anh both exhibited high level protein expression in 293T cells. Although the cleavage of H5cam appeared to be slightly better, the level of cleaved HA2 was comparable between H5cam and H5anh. Next, flow cytometry analysis was used to compare surface HA expression for H5cam and H5anh. Indeed surface expression of H5cam was significantly higher than that of H5anh.

A deletion of lysine residue was found at the cleavage site of H5anh when compared with H5cam. Swapping of HA2 domain (including the cleavage site) did not improve H5anh-pp production. Of notice, 8 AA residues were found different for H5cam and H5anh at the 130-loop and franking region of receptor binding domain (RBD). A series of H5anh mutants at the 130-loop region were generated for mutational analysis. Strikingly, all H5anh mutants with alanine to valine mutation at position 134, despite other sequence differences at 130-loop flanking region, largely restored the ability of H5anh to pseudotype lentiviral vector.

In conclusion, H5cam and H5anh showed similar level of protein expression in total cell lysates when transfected into 293T cells. However surface expression of H5cam was detected at a higher level than that of H5anh. This may partially explain the inability of H5anh to produce H5pp. Site-directed mutagenesis revealed that a single valine residue at position 134 of the 130-loop of RBD is critical for cell surface association of H5 HA and hence efficient H5pp production in 293T cells. It is likely that H5 HAs with alanine134 also confer higher receptor binding affinity; and consequently H5pp release from the producer cells is less efficient.

